# Linkages Between Geomagnetic Activity and Blood Pressure

**DOI:** 10.7759/cureus.45637

**Published:** 2023-09-20

**Authors:** Harvey N Mayrovitz

**Affiliations:** 1 Medical Education, Nova Southeastern University Dr. Kiran C. Patel College of Allopathic Medicine, Davie, USA

**Keywords:** earth magnetic field, space weather, heliobiology, solar activity, diastolic blood pressure, systolic blood pressure, geomagnetic activity, hypertension, solar storms, geomagnetic storms

## Abstract

This review aims to critically examine and present evidence for and against potential linkages between geomagnetic activity and its effects on blood pressure (BP). Four databases were searched for peer-reviewed papers written in English: PubMed, Web of Science, EMBASE, and Biomedical Reference Collection. Retrieved titles were first screened for potential relevance followed by an abstract review for further clarifications if warranted. The preponderance of the reported evidence is consistent with the concept that space weather and related events that cause sufficiently large changes in the geomagnetic field (GMF) can impact BP. The associated BP change in most but not all cases is one in which both systolic blood pressure (SBP) and diastolic blood pressure increase, with SBP appearing to be more consistently involved. The magnitude of the reported BP increase ranges from about 3 to 8 mmHg depending on the intensity of the geomagnetic activity. The initiation of these BP changes has been variably reported to occur shortly before the GMF change or in synchrony with the abrupt change in the GMF. Such GMF-linked BP changes are not present in all persons and there appears to be increased sensitivity in women and in persons with co-existing hypertension. The utility of these findings in assessing or treating persons with known or suspected hypertension remains to be determined via future research. Further, research directed at determining the factors that determine responders from non-responders to GMF changes is warranted.

## Introduction and background

The Earth’s magnetic field is influenced by its interaction with solar winds and other external events. When the solar winds are especially active, this can create geomagnetic disturbances that influence the underlying ionosphere resulting in “space weather” that impacts electric currents, plasmas, and even fields in Earth’s magnetosphere. Simply put, the Earth’s geomagnetic field (GMF) is the magnetic field surrounding the Earth [1-5].

The main component of this field is due to the circulation of Earth’s molten iron core and Earth’s rotation. It is sometimes referred to as the main field with an average value that ranges from about 25,000 nT to 65,000 nT depending on geographic location [1]. The field’s magnitude changes slowly largely dependent on internal Earth-bound processes. However, external sources can affect this field causing rapidly changing magnitudes that constitute geomagnetic disturbances. One such external source is attributed to changes in solar activity and solar wind via charged particle interaction with the main field including solar coronal mass ejections [2].

Solar activity varies according to an approximate 11-year cycle that depends on variations in the sun’s magnetic field [3]. When the sun’s magnetic field is at its maximum intensity and well organized, it contains the sun’s plasma in regions close to the sun’s surface. Over time, the field becomes less organized, and its ability to keep the high-energy plasma near the sun’s surface weakens. As a consequence, radiation in the form of bright solar flares may occur, sometimes associated with huge amounts of high-energy, high-speed charged particles that represent coronal mass ejections. When these disturbances occur, the terms geomagnetic storm, substorm, or pulsations may apply. If the changes induced in the GMF are sufficiently large, the term geomagnetic storm applies and is one type of disturbance thought to affect biological processes, even though the resultant change in the GMF may be of the order of 5%. In addition to these externally induced transient field changes, there are coexisting field changes mainly due to lightning radiant energy resulting in Schumann resonances [4]. The term heliobiology has also been used to describe possible space weather effects of solar and geomagnetic processes [5].

Mechanisms by which any of the various field alterations affect biological processes are mostly speculative with multiple hypotheses suggested. As a very broad summary of three possible groupings, there are (1) mechanisms triggered by rapid changes in the GMF, (2) mechanisms associated with resonant interactions triggered by electromagnetic fluctuations of various frequencies, and (3) mechanisms that trigger biological changes from space weather-induced changes in climatological parameters. In the following part of this introduction, a brief summation of the main aspects underlying geomagnetic disturbances is first presented as it is relevant to the following review. Thereafter, the main goal and emphasis of the review is to critically examine and present the evidence for and against potential linkages between geomagnetic activity and its effects on blood pressure (BP).

Main aspects underlying geomagnetic disturbances

The elements of the GMF of relevance herein are (1) the Earth’s magnetic field, (2) the concept of the global atmospheric electrical circuit, (3) Schumann resonances, (4) measurements and indices of geomagnetic disturbances, and (5) basic features of magnetic storms and their related geomagnetic disturbances. For readers who are already familiar with these aspects, skipping directly to the main review is a viable option. However, as terms, concepts, and other aspects used in the review are detailed in this section, it may be useful for others to examine this section.

Earth’s Magnetic Field

As noted, the Earth’s magnetic field has various sources but the most common and intense is the static magnetic field (SMF) generated by electrical currents within the liquid-iron outer core, referred to as the “core field” [6-9]. This action may be thought of as the rotation of charged molten iron generating a magnetic field that extends into space resulting in the magnetosphere. Other sources of the GMF include those attributable to magnetic minerals in the Earth’s crust and those small fields produced by the flow of seawater through the local magnetic field. The combination of all such sources comprises the internal field. The internal field is to be differentiated from external or disturbance fields, for example, those caused by atmospheric electrical currents due to lightning and other forms of planetary or solar disturbances.

Such time-varying external currents and their induced magnetic fields alter the internal fields. Thus, the observed magnetic field at a given location is a vector quantity that is time-dependent and mainly due to the sum of the core field, the field due to locally present magnetic rocks in the crust, and the field due to external disturbances. These disturbances produce changes in electrical currents that normally flow between the ionosphere and Earth within the global atmospheric electrical circuit (GAEC).

The Global Atmospheric Electrical Circuit

The GAEC is conceptualized as a spherical region of space extending from the Earth’s surface at one end to the ionosphere that starts at about 60 km above the Earth’s surface [10-12]. The boundaries of this nearly spherical cavity are highly conductive and in one sense can be visualized as a spherical capacitor filled with a leaky air dielectric through which electrical currents flow toward the earth and then toward the conductive ionosphere. This current flow is maintained at an electrical potential relative to the Earth’s surface in the neighborhood of 250 kV [11]. The generator for changes in these currents is largely dependent on thunderstorm activity. The magnitude of local currents at any altitude depends on the product of the local electric field and conductivity that itself increases with increasing altitude.

Another aspect of the GAEC is that it can support resonant electromagnetic waves with frequencies that depend on the dimensions of the cavity. For the GAEC, these frequencies may range between 7.8 and 45 Hz for resonance modes in the longitudinal direction and between 800 and 5,000 Hz in the transverse direction [13]. These phenomena are largely dependent on worldwide lightning activity and the energy it radiates [14]. The electric field generated by lightning strike radiation energy has a broad spectral content but mostly lies within the extremely low-frequency band (ELF = 3-3,000 Hz) and very low-frequency band (VLF = 3,000-30,000 Hz). The energy radiated by a lightning strike that enters the GAEC is reflected back and forth between the lower ionosphere and the Earth’s surface resulting in a propagated guided wave that travels with little attenuation from the location of its initiation. The composite wave is attributable to worldwide lightning strikes that occur with a frequency in the range of about 100 strikes/second. Depending on wave reflection aspects the resultant pattern may appear as a standing wave as in the Schumann resonance.

Schumann Resonances as Disturbance Fields

One major wave phenomenon that occurs with the most energy within the ELF band is referred to as Schumann resonances. These have a large spectral peak at a fundamental frequency of 7.8 Hz and have associated harmonics with minor peaks extending to and through 39 Hz and possibly having more harmonics [15]. These harmonic peaks occur at frequencies that correspond to wavelengths that approximate the Earth’s perimeter and are very much dependent on lightning-generated signals that vary in intensity diurnally, seasonally, and geographically [16]. The vertical electric field of the Schumann resonance fundamental is about 300 mv/m and the horizontal magnetic field intensity is of the order of 0.5 to 1.5 pT/Hz1/2 [17]. However, this background level may transiently increase to as much as 30 pT/ Hz1/2 due to cloud-to-ionosphere discharges.

It is noteworthy that experimental exposure of rat cardiomyocytes to a magnetic field at the Schumann resonance fundamental of 7.8 Hz impacted myocyte calcium kinetics, interpreted as a reduced calcium release from the sarcoplasmic reticulum, thereby impacting myocyte contraction features [18]. It is also interesting that measurements of the Schumann resonance field over a one-year interval have been reported to show a statistically significant association with cardiovascular-related hospital admissions in Granada, Spain [19]. In that study, analyses of clustered events were significant for directional components of the measured field with the north-south component being most relevant. A correlation between solar and geomagnetic activity and geomagnetic indices has also been described [20].

Assessing and Quantifying Geomagnetic Activity Changes

Studies that attempt to correlate a physiological or pathophysiological effect with changes in the GMF require a quantitative estimate of the magnitude of the field change. For this purpose, several parametric indices are available that are derived from measurements of the actual GMF and its temporal variations. The status of the GMF is often characterized by two planetary indices of geomagnetic activity (GMA) identified as Kp and Ap, both measured and reported in units of magnetic field strength and usually expressed in nT [21].

The planetary index Kp is calculated in a three-hour interval as the mean of K-indices determined at 13 worldwide geomagnetic observatories located between 44° and 60° North and South geomagnetic latitudes. Ap is the overall daily index of geomagnetic activity determined as the mean of eight three-hour values from the same observatories sometimes referred to as k-sum. The diurnal variation of the naturally occurring, time-varying magnetic field is measured by each local geomagnetic observatory as the K index. The value of the Kp index is used as a measure of the planetary GMF effect that includes all currents and magnetic deviations they produce on the ground. Kp quantifies disturbances in the Earth’s magnetic field on a scale of 0-9, with ≤2 indicating low GMA and ≥5 indicating a geomagnetic storm [22-24]. These are logarithmic ratings that are related to the absolute change in the Earth’s magnetic field, although attempts have been made to extrapolate the global Kp values to the local situation [25]. GMA can also be measured using the Ap index which is defined as the earliest occurring maximum 24-hour GMA value obtained by computing an eight-point running average of successive three-hour Ap indices during a geomagnetic storm event and is uniquely associated with the storm event.

Another index, felt by some to be superior to planetary indices in some studies, is the hourly storm time disturbance index (DST) [26]. The DST is an hourly index of low-latitude magnetic activity obtained from magnetometer measurements at four observatories located near the magnetic equator and is an indicator of magnetic storm intensity [27,28], although with some limitations [29]. Its value is expressed in nT and is proportional to the total kinetic energy of sun-originated high-energy particles present within the outer band of the Van Allan belt and is a suitable measure of the magnetic storm’s intensity. Geomagnetic storms are stratified by the amount of reduction in the DST value.

Reductions in DST <30 nT are considered to represent a weak storm, between 30 and 50 nT as a moderate storm, between 50 and 100 nT as a strong storm, between 100 and 200 nT as a severe storm, and more than 350 nT as great storms [30]. Typically, storms have three phases characterized by a sudden start, followed by a rapid decay, and recovery to the prior “quiet time” level.

Another index is the SYM-H geomagnetic index which has a time resolution of about one minute compared to the one-hour resolution of the DST. The SYM-H is defined as the baseline-corrected, magnetic latitude-adjusted average of the disturbance component at each minute for six geographically separated recording stations. [31]. An additional parameter that has come into use is the ultra-low frequency (ULF) wave index that characterizes the ground-level dynamic electromagnetic field changes in the frequency range of 0.001 Hz (1 MHz) to about one MHz [32]. This index is sensitive to the turbulent nature of electrodynamic changes associated with solar wind-magnetosphere-ionosphere interactions and changes and can be a better index than some of the planetary indices [33].

Magnetic Storms and Physiological Impacts of Solar Phenomena

**Figure 1:**
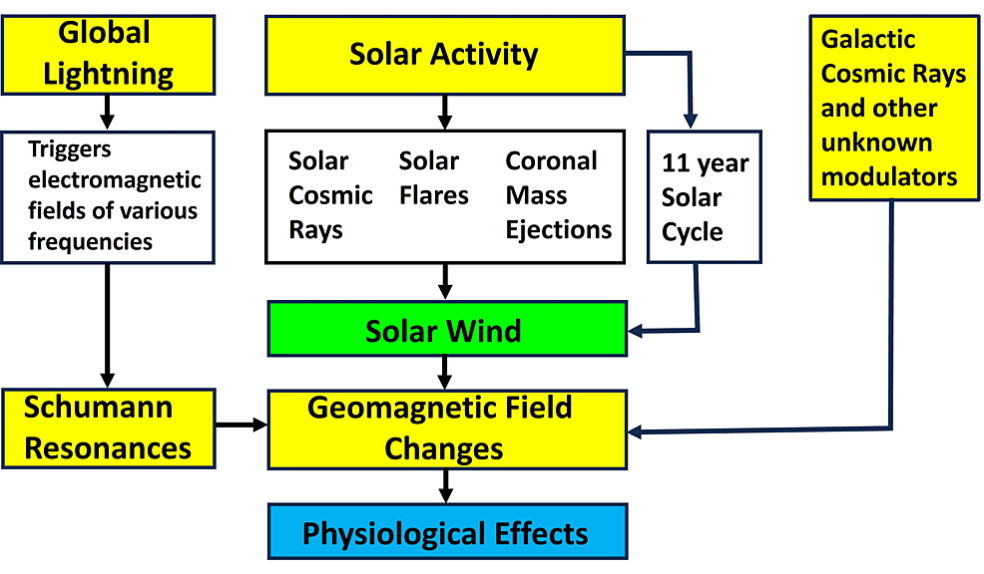
Some main contributors to changes in the geomagnetic field (GMF). The schematized version of the processes impacting the GMF from solar and other sources is an overview and not meant to be fully inclusive of all processes that might alter the GMF. The coupling mechanisms between changes in the GMF and associated physiological effects are unresolved. The physiological effect of interest in this study is the effect on blood pressure. Figure courtesy of Dr. HN Mayrovitz.

Naturally occurring geomagnetic disturbances are thought to be associated with biological and clinical events [34]. Solar flares and solar activity generate magnetic storms, which through modulation of the solar wind, cause geomagnetic anomalies in the Earth’s environment impacting living organisms [34-36]. During storms, changes in the magnetic field, particularly small pulsations of the magnetic field accompanying the geomagnetic storm, have been reported to affect human health adversely, with the heart and cardiovascular system being the main targets [37]. Cardiovascular exacerbations have been reported to correlate with high negative values of the interplanetary magnetic field component and the geomagnetic disturbances they induce. Cardiovascular-related changes reported range from changes in blood’s properties including red cells [38], and white cells [39] to changes in BP [40] discussed later in detail. An indication of the possible impact on BP in hypertension is suggested by the reported increase of 12% in ambulance calls for hypertension-related matters [41]. A summary of some of the main contributors to changes in the GMF is schematized in Figure 1.

The mechanisms by which solar activity influences biological systems through variations of the GMF are still being identified. It has been proposed that geomagnetic pulsations with a period of seconds to minutes are important determinants of geomagnetic biological disturbances [37]. Because the state of a GMF can vary within 24 hours from a quiet state to a geomagnetic storm, the results of studies can depend on the timing of measurements [34]. These magnetic storms are characterized by large disturbances in Earth’s GMF that as noted are often caused by increased solar wind activity effects. This can include the ejection of solar and coronal mass that is propelled into space toward Earth. The augmented flux of high-energy particles impinging on the magnetosphere is associated with a reduction in the Earth’s magnetic field, usually affecting the horizontal component of the magnetic field (H-field). As noted, the intensity of these storms is often assessed by the magnitude of the change in DST with reductions ≥100 nT considered intense storms [28] and reductions ≥250 nT considered super intense storms by some studies [27].

The potential impacts of magnetic fields on cardiovascular parameters have often been studied via laboratory experiments in which animals were exposed to magnetic fields of various intensities and the impacts of such exposure on BP, heart rate, and blood flow are evaluated. Major contributions are attributable to the pioneering work of Gmitrov, Ohkubo, and Okano and their co-workers who studied the effects of SMF on BP [42-48] and skin microcirculation [49-53], among other aspects. Their findings add to the literature regarding the suspected possible impacts of GMF on these same parameters. However, the focus of the present study is on the potential linkage to blood pressure.

## Review

This review aims to critically examine and present the evidence for and against potential linkages between geomagnetic activity and its effects on BP.

Search strategy

The following four databases were searched for peer-reviewed full papers written in English: PubMed, Web of Science, EMBASE, and Biomedical Reference Collection: Comprehensive. Within each database, the term “blood pressure” was required to be present anywhere within the article text. This criterion was combined with the following logical “AND” condition applied to the article title: “geomagnetic*” OR “space weather” OR “heliobiology” OR solar* OR “Schumann” OR “lightning” OR “magnetic field*” OR “cosmic rays”. The Asterix (*) served as a wild card.

Retrieved titles were first screened for potential relevance followed by an abstract review for further clarifications if warranted. Articles that were deemed relevant were retrieved and reviewed. In most cases, the bibliography of the retrieved articles provided additional sources with supplemental searches done as needed.

Geomagnetic linkages to blood pressure that included hypertensive subjects

A short letter to the editor more than 25 years ago described ambulatory BP measurements in hypertensive patients that appeared to be consistent with a potential impact of high versus low geomagnetic activity [54]. In that study, systolic blood pressure (SBP), measured in 39 patients on high k-value days, exceeded that measured in 35 other patients who were assessed on quiet k-index days. The reported increase was on average 7 mmHg. However, as the same patients were not measured on both quiet and stormy days, the findings are inconclusive. Still, focusing on patients with hypertension, a small study of eight patients with isolated systolic hypertension suggested a potential gender difference in BP responses during a five-day self-monitoring protocol during a magnetic storm with hypertensive men more sensitive to changes in the GMF than women [55]. In this study, daily BP data, measured every 30 minutes, was averaged, and individual and composite trends of SBP and diastolic blood pressure (DBP) were evaluated against the daily H-field. This reported gender difference was opposite from that reported earlier in a group of 51 young normotensive subjects (33 women) [56].

A more extensive and controlled study retrospectively evaluated 447 non-treated hypertensive patients (181 females/266 males, aged 21 to 85 years) whose BP was measured at least every 30 minutes via diagnostic ambulatory BP measurements [57]. The K-sum index was obtained at an observatory 287 km distant from the BP measurement. The correlation between the 24-hour BP average and the K-sum index on the day of BP measurement, and the two prior days, was evaluated. The main finding was a weak but statistically significant positive correlation between both systolic and diastolic BP and the K-sum on the day of the BP measurement. No significant correlation was found for the two days before the BP measurements, suggesting that the correlations were not spurious. Correlations ranged between 0.109 and 0.150, The authors speculated that the positive linkage might be due to increased stress-related processes experienced during higher K-sum days.

Additional evidence for a dependence of BP in humans on the intensity of the GMF disturbance was reported as an increase in SBP and DBP from one day before to two days after a geomagnetic storm in 86 persons evaluated during the Spring of 2001 and Autumn of 2002 [40]. The studied group consisted of 53 females and 33 males with an average age of 47.8 ± 11.9 years, of whom 22 had hypertension. These investigations conducted by Dimitrova and her colleagues provided significant additional information regarding the impacts of geomagnetic factors on BP. In analyses reported in 2004 [40,58], the measured SBP and DBP values were subjected to correlational analysis against the planetary indices (Ap and Kp) as well as variations in the local GMF (H-field). In their analyses, which had data for 92 days, the amplitude of the change in the H-field was used to categorize the intensity of GMA. In this categorization, a change of 35-70 nT was a disturbance, 70-120 nT a weak storm, 120-200 nT a moderate storm, 200-320 nT a major storm, and >320 nT a severe storm. A somewhat similar categorization was used for a combined grouping of Ap and Kp indices in which an Ap index of ≥100 constituted a severe storm. 

Results indicated small correlations between SBP and DBP vs. geomagnetic indices ranging from 0.11 to 0.15. However, when a four-factor multivariate analysis of variations was used, a main effect showing a statistically significant increase in both SBP and DBP with increasing geomagnetic activity was reported (p < 0.01). It was reported that during local geomagnetic storms both SBP and DBP increased in about 91-92% of subjects. The overall average BP increase was reported as 12% with the highest BP value occurring on the day of a severe storm and the amount of BP increase related to the amount of increase in geomagnetic activity. Comparing SBP and DBP values between the least and most active geomagnetic activity demonstrated increases of 6-8 mmHg. The composite data indicated that the highest BP value occurs on the day of the severe storm with an increase of about 15 mmHg in SBP and about 8 mmHg in DBP compared to pressures measured during a low-intensity GMF disturbance.

Reports of further analysis among the same group of subjects indicated that women tended to be more sensitive to the effects of the geomagnetic activity, and persons taking antihypertensive medications were also more reactive [59]. Later analysis of these subjects indicated SBP and DBP increased with increments of geomagnetic activity, as measured by changes in either Ap or DST indices [60].

An additional study of this group in terms of the effect of cosmic ray intensity decreases of between 3.5 and 9.5% during the same seasonal intervals demonstrated significant SBP and DBP increases by an amount that increased with decreasing cosmic ray intensity [61]. Average increases in both were about 11% from the least to greatest cosmic ray increase. In an attempt to account for observed pre-storm BP effects, it was speculated that pre-storm extremely low-frequency pulsations may be involved [62]. Relevant to this pre-storm effect is the fact that the radiation associated with the mass ejection takes only about eight minutes and 20 seconds to reach Earth whereas the solar wind and its effects that cause the geomagnetic storm take one to two days to occur.

Geomagnetic linkages to blood pressure in normotensive subjects

Animal Laboratory Studies

A series of experiments suggested a potential role of geomagnetic linkages to baroreceptor function as a BP modifier using a rabbit model [36,63-68]. Based on prior work that suggested an SMF as low as 0.2 T applied to the carotid sinus area could reduce BP [42], the potential modulating effect on SMF-induced BP reductions by the GMF was evaluated [69]. In these experiments, the animal’s BP was increased via infusion of vasoconstrictive substances, and then the hypotensive effects of the applied SMF were evaluated on low and high k-index days. Results suggested that high K-index days were associated with a greater SMF-induced decrease in diastolic and mean BP than occurred on low K-index days.

Other studies using the rabbit model, focused on determining the effects of the GMF as a modulator of carotid baroreceptor sensitivity (BRS). This was done using bolus infusions of either a vasodilator to decrease BP or a vasoconstrictor to increase BP. After infusion of the vasoactive substance, BRS was evaluated as the change in heart rate for a change in BP [63]. The potential impact of geomagnetic activity was assessed by grouping data from experiments done on low K-index (0-1) versus high K-index (2-5) days in which K-index values were obtained from a nearby observatory. The main result suggests a decrease in BRS associated with increased K-index. This decrement in BRS is to be distinguished from increased BRS sensitivity reported to be present when rabbit carotid baroreceptors were exposed to a 0.5 T SMF of a magnet for 45 minutes [70]. Further experiments, in which the carotid sinus was exposed to an SMF of 350 mT, also demonstrated a significant positive correlation between BRS and microcirculatory blood flow [68]. However, the augmented blood flow was reported to diminish in proportion to the prevailing K-index. As a consequence, increased GMA significantly attenuated both blood flow and BRS in the rabbit model. Based on a serendipitous occurrence of a magnetic storm during the non-invasive measurement of SBP in rats, it was reported that there was an increase in SBP on the first day of the storm [38]. These researchers reported that this SBP increase could be replicated in rats using artificially created magnetic fields simulating the naturally occurring phenomena.

Human Laboratory Studies

A well-controlled laboratory study in which nine young men were exposed to simulated GMFs that mimicked quiet or stormy days failed to demonstrate an impact on either SBP or DBP [71]. However, a reduction in nailfold capillary blood velocity was noted to occur, especially in the evening hours. Another small study in which three healthy men were exposed to an artificial field mimicking the amplitude (50 nT) and frequency (1.6 MHz) of a geomagnetic storm also failed to find changes in BP [72]. In this study, exposure was during 12 consecutive weekends with BP measured every 30 minutes during exposure.

Natural Environment Geomagnetic Field Effects

A large study evaluated SBP and DBP of 152 men and 152 women over a six-year interval and reported a correlation between BP and the H-field mainly apparent during nighttime [73]. It appeared that these changes were more dominant in women as compared to men. The impact of geomagnetic factors on SBP, DBP, and heart rate has been described as dependent on the prevailing atmospheric temperature, humidity, and, most importantly, atmospheric pressure [74]. In this study, 197 healthy young persons reported their BP and heart rate for seven consecutive days. These values were then compared with Kp values and other atmospheric parameters. After a rigorous analysis, the authors concluded there was a small but highly significant association of the Kp value with an increase in SBP (r = 0.39, p =0.005) and heart rate (r = 0.33, p - 0.006) and a decrease in DBP (r = -0.23, p = 0.0008). Based on these reported trends, there would be an increase in pulse pressure (SBP - DBP) with increasing Kp values, although this is yet to be studied experimentally.

Schumann Resonance Findings

In contrast to the reported BP increase with increasing Kp, decreases in SBP and DBP were observed on days of enhanced versus normal Schumann resonance activity [4]. These findings were based on 24-hour ambulatory BP measurements in 56 adults (30 males) made over seven consecutive days with Schumann resonance values obtained from a nearby station and classified as enhanced or normal Schumann resonance activity days with enhanced activity set as an amplitude greater than 1.97 pT. Although there was a significant difference in group means on enhanced versus normal Schumann resonance days, the difference was small amounting to 2.9 mmHg systolic and 1.9 mmHg diastolic with a 2.2 mmHg reduction in mean arterial pressure. The overall changes were dominated by the 32% of participants who demonstrated a BP reduction.

Geomagnetic and Weather Factors

An expansion of our understanding evolved from investigations of the combined effects of meteorological weather, space weather (geomagnetic), and solar parameters on DBP [75,76]. A weather strength parameter (S) was devised that included temperature (T), wind speed (WS), cloudiness (C), humidity (RH), atmospheric pressure (P), and visibility (V), with S expressed as S = [(2 + 100T) × (1 + 10WS) × (10C + RH)]/(P2 × V). By a curve-fitting approach, they determined the parameter set to achieve the best correlation to account for the observed DBP increase. This was achieved using a parameter that included Kp, S, the ULF wave index, and the sunspot number (SSN) that considers the number of individual sunspots as well as groups of sunspots. With this formulation, the DBP was directly dependent on Kp and the product of ULF and SSN and inversely related to S. The maximum correlation was 0.65 during the Autumn monitoring interval and 0.50 during the Spring. The potential dual role of weather and geomagnetic impacts on BP was also evaluated in a group of 27 healthy women who had their morning BP measured in triplicate on working days for 13 contiguous months [77]. The triplicate values were averaged to arrive at daily SBP and DBP values. One main result of the analysis indicated that both SBP and DBP were positively correlated with increasing Kp values and decreasing atmospheric temperature. Based on individual subject correlations, 85% of subjects had a statistically significant negative correlation between SBP and atmospheric temperature, whereas 44% of subjects had a statistically significant positive correlation between SBP and Kp. Similar percentages were observed for DBP (77% and 44%). It is noteworthy that the temperature range over the 13-month observation interval ranged between -20°C and +20°C.

More recently, an extensive analysis of the potential impacts of solar activity-related changes in the GMF on SBP and DBP in 675 elderly men was reported [78]. Sunspot numbers, Kp, and values of the interplanetary magnetic field (IMF) were used in a multivariate mixed model to assess impacts on BP over a 28-day window. The overall findings indicated small (2.7 mmHg) but statistically significant BP increases that achieved the greatest value 28 days after the initial geomagnetic storm exposure.

Small Numbers of Subjects with Longitudinal Studies

In a unique study of the role of space weather on BP and heart rate, data from a single person was recorded and analyzed over an interval of 16 years resulting in the accumulation of about 83,000 data points that encompassed 27 magnetic storms [79]. Using the SYM-H index as a measure of geomagnetic activity a temporary increase in SBP and DBP was indicated just before the storm initiation. Only heart rate showed a sustained decrease after storm initiation, with the heart rate reduction becoming statistically significant after about eight hours and remaining decreased for the storm duration. A similar long-term study but on only a few subjects was undertaken in nine healthy subjects with an age range of 42-52 years. SBP and DBP were measured in the morning on working days during a three-year interval [80]. No significant correlation was detected between BP and Kp over this interval; however, a BP peaking was observed one to two days after a geomagnetic storm in a few subjects. A single-subject study in which pulse wave velocity was measured together with the GMF for three years reported pulse wave velocity to be increased as a reflection of an increase in arterial stiffness [81].

Study limitations

A potential limitation is that only literature published in English was included in this review. Russian and Eastern European researchers have made important contributions to the field [5,62,74,76,82], but their articles that were not otherwise translated into English were not included. However, a post-hoc literature search using the same criteria as originally used, except for using the Russian language, yielded only eight non-duplicate papers. After reviewing the English abstracts, these were found to either not be relevant or have results that were already translated and included in the present review. However, there may still have been undiscovered studies not found in the databases herein used.

## Conclusions

The preponderance of the reported evidence is consistent with the concept that space weather and related events that cause sufficiently large changes in the GMF can impact BP. The associated BP change in most but not all cases is one in which both SBP and DBP increase, with SBP appearing to be more consistently involved. The magnitude of the reported BP average increase may be as low as 3 mmHg but ranges from about 3 to 8 mmHg depending on the intensity of the geomagnetic activity. The initiation of these BP changes has been variably reported to occur shortly before the GMF change or in synchrony with the abrupt change in the GMF. Such GMF-linked BP changes are not present in all persons and there appears to be an increased sensitivity in women and in persons with co-present hypertension. The utility of these findings in assessing or treating persons with known or suspected hypertension remains to be determined via future research. Further, research directed at determining the factors that determine responders from non-responders to GMF changes is warranted.
